# Adaptation of the SAGEFS scale on attitudes toward gender equality in football in the international school context

**DOI:** 10.3389/fpsyg.2023.1265021

**Published:** 2024-01-11

**Authors:** Luz Marina Méndez-Hinojosa, Pedro Gil-Madrona, David Zamorano-García, Juan Ángel Simón-Piqueras, Juan Carlos Padierna-Cardona, Elizabeth Flores-Ferro, Martín Francisco González-Villalobos, Ratko Pavlovic, Fernando Maureira-Cid, Natalia Gómez-Santos, Sonia Morales-Calvo, Magaly Cárdenas-Rodríguez

**Affiliations:** ^1^Departamento de psicología. Facultad de psicología, Universidad de Nuevo León, Monterrey, México; ^2^Faculty of Education of Albacete, University of Castilla-La Mancha, Albacete, Spain; ^3^Facultad de Educación Física, Recreación y Deporte, Institución Universitaria Politécnico Colombiano Jaime Isaza Cadavid, Medellín, Antioquia, Colombia; ^4^University of Bernardo O’Higgins, Santiago, Chile; ^5^University Health Sciences Center, University of Guadalajara, Guadalajara, Mexico; ^6^Faculty of Physical Education and Sports, University of East Sarajevo, East Sarajevo, Bosnia and Herzegovina; ^7^Departamento de Educación Física, Deporte y Recreación, Universidad Metropolitana de Ciencias de la Educación, Chile, Santiago, Chile; ^8^Faculty of Social Sciences and Information Technologies of Talavera de la Reina, University of Castilla-La Mancha, Talavera de la Reina, Spain

**Keywords:** instrument development, soccer, children’s sports, gender, physical education, equality sport

## Abstract

The objective of the present study is to evaluate the psychometric properties of the Scale of Attitudes toward Gender Equality in Football in the Context of Schools (SAGEFS) in the international context. This sample was formed by *N* = 6,101 students. The study was conducted by applying the SAGEFS. The model of the three factors correlated in the complete sample and for each country was correlated using AFC. The structural model was appraised by employing eight indices: the relative Chi-squared index; the goodness of fit index and its adjusted formula; the normal fit index; the comparative fit index; the standardized Quadratic Mean; and the Quadratic Mean Error of Approximation. To conclude, the results evidence the presence of psychometric properties that are indispensable for the measurement of attitudes toward gender equality in the context of schools.

## Introduction

Mixed-gender education currently pre-dominates in most countries. However, coeducation is not that simple and requires an educational approach. In this respect, it is also possible to add aspects related to the socio-psychological dimension and several other variables, such as egalitarian beliefs, sexist beliefs, and the perception of sexist relationships. Coeducational education is not only the transmission of content that the student must learn, it also affects other constructs and attitudes along with the social structures involved (Valdivia-Moral et al., 2015).

In the case of teaching staff, Physical Education (PE) teachers do not provide real alternatives to combat the gender prejudices and inequalities present in sexist relationships to spread equality at school. This has been explained in studies carried out in several continents (Castillo et al., 2012; Valdivia-Moral et al., 2012, 2015, 2018).

For example, Soler (2009) and Sánchez-Hernández et al. (2018) indicate that soccer is one of the subjects taught with a higher gender culture load. This gender load remains dormant as regards PE classes about aspects such as space, materials, kit, language employed, feedback, evaluation, group formation, etc (Soler, 2009; Valdivia-Moral et al., 2018). It is, therefore, important that this material and content such as soccer are employed to work on equalitarian beliefs, which can be understood as the set of ideological principles of a person or group that promote the treatment of men and women, of the activities that they carry out, and of their achievements in an egalitarian way.

For this reason, it is important to teach pupils about gender equality in different knowledge areas (Brown, 2018). As Joy and Vasanthan (2022) explained, feminism fosters the erasing of the social discrepancy between what the patriarchy expects from women and reality. Due to this, education is the key to understanding the reality around us and the different social needs that might appear due to gender inequality (Joy and Vasanthan, 2022). The same was reported by Gaytán and Basso (2022), whose study included gender stereotypes and inequalities regarding not only gender but also ethnicity and sexuality. Men and women encounter many differences in sports because of the existence of stereotypes that create barriers and problems. All these problems are affected by a toxic masculinity that builds false expectations, which are reflected in violence against all collectives and practices, such as sports (Gaytán and Basso, 2022).

Upon analyzing the role of women in soccer, Woodhouse et al. (2019) and Ramírez and Restrepo (2018) recently showed that the participation of female players is much lower than that of their male counterparts, despite the increase in female players taking part in this sport (FIFA Activity Report, 2018). Although advances have been made regarding equality between men and women in soccer, many gender stereotypes and sexist beliefs continue to exist in this discipline. This is, in large part, because there are still many countries and cultures in which women do not have the same rights as men. At this point, it is important to highlight that those sexist beliefs are practices or attitudes that foment the different treatment of people according to their gender. In the case of soccer, this leads to a situation in which more importance is placed on the achievements of men, such that women’s soccer has less impact. The study by Olmedilla et al. (2021) indicates that male youth soccer players manage the stress of competition and the evaluation of their performance better, and youth soccer players have higher scores in team cohesion than the boys.

As explained by Vannini and Fornssler (2011), there is also a problem related to women in sports: over-sexualization. Women in sports have long been sexualized because of their clothes or gestures and, depending on the sports they play, they are more sexualized than other women. For this reason, it is important to empower women to play sports, educate themselves, and be active no matter what (Mercer-Mapstone and Mercer, 2018).

Both egalitarian and sexist beliefs play a highly important role in physical activity, specifically in the case of soccer, since they promote situations in which girls do not participate in physical activities and do not, therefore, obtain the benefits that have been reported at a psycho-socio level (Bustamante-Sánchez and Del Coso, 2020). This has a direct consequence on the way they perceive their relationships with members of the opposite gender. In the case of girls, this perception is determined by the influence of social construction on body identity and the role that education, sports and health have on it (Walseth et al., 2017). In the case of boys, their perception of sexist relationships in this sport tends to be influenced by the idea that soccer is an activity that reinforces masculinity. Another key aspect to highlight is that although women have been excluded from practicing sports until very recent times, they have managed, in recent years (Salido-Fernandez, 2020), to challenge gender stereotypes to obtain a greater presence in a masculinized environment in both the elite (Sainz de Baranda, 2014) and the amateur field.

There is currently a lack of studies that analyze the role played by girls in soccer, along with their relationship with playing the game and with their male companions. The studies carried out by Ramírez and Restrepo (2018) in Latin America and Woodhouse et al. (2019) in Europe regarding the role played by women in soccer, therefore, stand out in this respect. Nevertheless, the studies by Sánchez-Hernández et al. (2018) and Soler (2009) in Spain carry out a more detailed analysis of what occurs in PE classes. This consequently shows that it is necessary to develop studies in different countries in order to be able to compare different cultures.

Upon focusing on the creation of instruments with which to evaluate equality in physical activity, it is possible to find tools such as that developed by Valdivia-Moral et al. (2015), which is intended for use within the teaching profession, or that of García et al. (2010), which evaluates students’ attitudes toward equality in PE. The questionnaire concerning gender beliefs and stereotypes regarding physical activity and sport (GBSFAS) developed by Granda et al. (2018) is, meanwhile, more focused on the sphere of sport. After reviewing what the scientific literature presents in relation to this matter in question, the conclusion was reached that there are no instruments with which to evaluate attitudes related to gender equality in people of school age. Moreover, it is important to bear in mind that gender inequality and social exclusion might start at high school due to stereotypes based on gender, such as the ability to practice sports, to compete, and to be part of a sports team (Mercer-Mapstone and Mercer, 2018).

Keeping the current situation in mind, along with the state of the aforementioned variables, the objective of the present study is to adapt the Scale of Attitudes toward Gender Equality in Football in the School Context (**SAGEFS**) to the context of Spain, Ukraine, Morocco, Iran, Bosnia, Mexico, Colombia, Chile, and Brazil.

## Materials and methods

### Participants

The study was developed using a non-probabilistic sample. This sample comprised *N* = 6,101 students, 49.5% of whom were male and 50.5% of whom were female. The participants were an average of 13.31 years of age, CI 95% [13.25, 13.34], with the youngest being 9 and the oldest being 20. The mean age of the male students was 13.17 (SD = 2.323), while that of the female students was 13.45 (SD = 2.369). Statistically significant differences were found upon comparing the ages of the male and the female students: *t* = −4.714, *p* < 0.001. The mean regarding the perception of playing soccer well was 5.31 (SD = 2.70). The mean for the male students was 6.20 (SD = 2.521), while that for the female students was (SD = 2.585). Statistically significant differences were found upon comparing the perceptions of male and female players playing soccer well: *t* = 26.848, *p* < 0.001.

With respect to the students’ nationalities, 15.3% were from Spain, l 5.8% were from Ukraine, 6.2% were from Morocco, 17% were from Iran, 14.8% were from Bosnia, 9.3% were from Mexico, 7.9% were from Colombia, 17.7% were from Chile, and 6.1% were from Brazil. Statistically significant differences were found upon comparing the average age of all the nationalities: *F* = 297.911, *p* < 0.001.

The frequencies and descriptive statistics of the socio-demographic and anthropometric variables are shown in Table 1.

**TABLE 1:** Frequencies and descriptive statistics of sociodemographic and anthropometric variables.

		*All*		*Age*		*Weight*		*Height*	
		*N*	%	*M*	*SD*	*M*	*SD*	*M*	*SD*
Country	Spain	933	15.3	10.83	0.881	42.60	9.451	150.37	9.148
	Ukranie	351	5.8	13.40	2.149	52.00	13.256	162.69	13.165
	Morocco	376	6.2	13.06	2.128	50.89	13.018	159.32	12.075
	Iran	1036	17	12.67	1.959	52.00	0.000	155.00	0.000
	Bosnia	900	14.8	14.00	2.581	57.31	14.434	168.21	12.280
	Mexico	566	9.3	14.81	2.144	55.55	13.173	159.67	14.081
	Colombia	482	7.9	13.42	2.145	49.79	15.483	150.71	15.676
	Chile	1082	17.7	14.40	1.871	55.09	10.747	160.27	11.149
	Brazil	375	6.1	14.19	2.091	53.58	11.708	159.44	10.578

N, number of cases; M, arithmetic mean; SD, standard deviation.

### Instruments

The study was carried out by applying the SAGEFS, whose design and evaluation of psychometric properties were created for Spain. This scale includes 20 positive items with 5 Likert-type response options, ranging from “totally disagree” (1) to “totally agree” (5). The scale has three dimensions: (1) egalitarian beliefs, (2) sexist beliefs, and (3) perception of sexist relationships. The product of the adaptations and psychometric analyses in the nine countries in which the scale was validated was the Short Scale of Attitudes toward Gender Equality in Football in the Context of Schools (SSAGEFS), which contained 11 items [egalitarian beliefs (4 items), sexist beliefs (4 items), and perception of sexist relationships (3 items)].

Perceived motor competence was measured using the Spanish version of the AMPET [Achievement Motivation in Physical Education test (Pérez et al., 2015)]. This factor was composed of seven positive items, with five response options ranging from ‘totally disagree’ to ‘totally agree’ on a Likert scale of 1 to 5. The internal consistency index obtained after its application was α = 797.

### Procedure

To carry out this study, the coordinators contacted renowned researchers in various countries to inform them about the study and discover their interest in participating in it. In this way, the application of the scale to the context of each country was programmed, counting on those researchers who showed interest in participating in the study. For this, translators were hired, who translated the scale into the language of each country where it would be applied using the back translation method, which is a translation verification tool involving translating text into the desired language and then back into its original language. For this activity, the translators were asked to use a synonym or idiom if they identified any word that was not familiar in the context where the instrument would be applied. It is necessary to mention at this time that this article only intends to analyze the construct validity and reliability of the instrument for its adaptation to the international context; since the content validity was done in a previous study (Gil-Madrona et al., 2022) and the purpose of the present study was to adapt the SAGEFS scale regarding attitudes toward gender equality to the international school context. However, in accordance with Ortega et al. (2008), who, as part of the content analysis, established the judgment of experts and the understanding of the items by pilot sample, the procedure was applied to a sample of five subjects in each country; this sample had the same characteristics as the final sample. Each researcher participating in the study (one from each country) told the subjects to read the items carefully, answer them, and indicate if they had questions, doubts, or any comments about the writing or understanding of the instrument (qualitative degree of understanding of the test). All information was recorded in detail. Next, they were asked to write “1” for each item if the item could be understood within a single reading and 0 otherwise (quantitative degree of understanding). Based on the subjects’ comments, corrections were made to the items. Regarding the quantitative values and the intercoder reliabilities, where the intercoder reliability was 0.85 or more, the comments made by the subjects were taken into account.

The schools that stated their intention to participate were given a training course via Skype in order to provide a detailed explanation of the objective of the research, what the study would consist of, the instructions and protocols required to utilize the questionnaires in each of the educational institutions, the databases to which they would have to upload the data eventually obtained, and informed consent forms that would have to be filled out by the families and the schools of those participating in the research. The aforementioned information was also sent in written format to those responsible in each of the participating countries.

Before handling the questionnaire, written permission to do so was obtained from the academic authorities corresponding to each of the schools in the countries studied. The students were invited to take part in the study on a voluntary basis, and a parent or tutor signed the corresponding informed consent form for underage participants. Then, the students were provided with an explanation of the object of the research, along with the relevant instructions, and were given the questionnaires. They did not receive any type of economic, material, or academic recompense for their participation. All the data were obtained in accordance with the ethical guidelines laid down in the Helsinki Declaration.

### Data analysis

The model of the three factors correlated in the complete sample and for each country was correlated using CFA. The structural model was appraised by employing eight indices: the relative chi-squared index (χ2/df), the goodness of fit index (GFI) and its adjusted formula (AGFI), the normal fit index (NFI), the comparative fit index (CFI), the standardized quadratic mean (SRMR), and the quadratic mean error of approximation (RMSEA). Values of χ2/df ≤ 2; GFI, NFI, and CFI ≥ 0.95; AGFI ≥ 0.90; and SRMR and RMSEA ≤ 0.05 were considered to show a good fit, while values of χ2/df ≤ 3; GFI, NFI, NNFI, and CFI ≥ 0.90; AGFI ≥ 0.85; SRMR ≤ 0.10; and RMSEA ≤ 0.08 were considered to indicate an acceptable fit. The equivalence of the goodness of fit was verified by testing the difference between the chi-squared and relative differential chi-squared statistics (Δχ2/Δdf), and the difference between CFI, NFI, and NNFI. Values of p > 0.05 for H0: Δχ2 = 0, Δχ2/Δdf < 2, Δ CFI, ΔNFI, and ΔNNFI ≤ 0.01 were considered to show the equivalence of the goodness of fit (Byrne, 2016).

The internal consistency of the three-correlated-factor model was verified by employing McDonald’s coefficient (ω > 0.70; Raykov and Hancock, 2005). The normality fit of the description of the distribution of the SAGEFS was contrasted by employing the Kolmogorov-Smirnov test with correction for Lilliefors significance correction. The multivariate normality of the structural modal was determined by employing the Mardia coefficient, which should be ≤ 70 (Rodríguez and Ruiz, 2008).

The means per gender were compared by employing the Student’s t statistic for independent samples. This parametric test was utilized despite the lack of a normal distribution owing to the fact that it is robust when this supposition is not fulfilled when the sample size is large (Poncet et al., 2016). The effect size (ES), as regards the difference in the means, was estimated by employing the Hedges g statistic, whereby values higher than 0.20 are considered to be a small ES, those of 0.40 are medium ES, and those of 0.80 are large (Hedges, 1981).

The concurrent validity was verified using Pearson’s correlation coefficient. Since the normality was not fulfilled, the confidence intervals were calculated using the repetitive sample with the simulation of 2,000 random samples created using the percentile method (Bishara and Hittner, 2015).

## Results

In the nine participating countries, the application of the scale to a pilot sample indicated that its application was viable.

### Validity and reliability of the three-correlated-factor model

**FIGURE 1:**
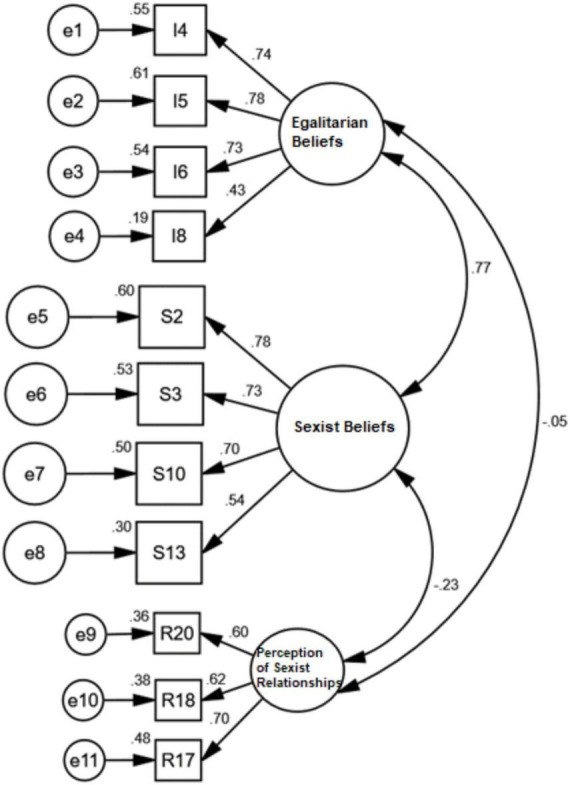
Model for the SAGEFS.

The three-factor model was specified using its independent measurement residues. Table 2 shows the fit of the three-correlated-factor model to the complete sample and by country. With regard to the general sample, the fit was good for seven of the eight indices employed (GFI, AGFI, CFI, NFI, NNFI, RMSEA, and SRMR), although the relative chi-squared value was high (χ2/gl = 15.637). However, this index rarely obtains acceptable values for large samples (Barrett, 2007). Moreover, the factorial weights varied between 0.43 and 0.78 and were, therefore, greater than the minimum required value of 0.40 (Williams et al., 2010). The mean λ was).634, which was close to the desired value of 0.70 (Hair et al., 2014). Please note that it was necessary to eliminate nine items in order to improve the indicators of fit. The model obtained for the SAGEFS is shown in Figure 1.

**TABLE 2:** Goodness of fit indices for each sample and total sample.

Indices	Total	Spain	Ukraine	Morocco	Iran	Bosnia	Mexico	Colombia	Chile	Brazil
χ^2^	641.106	135.249	105.451	84.007	−	169.763	108.623	50.816	115.149	74.100
*df*	41	41	41	41	−	41	41	41	41	41
*p*	< 0.001	<0.001	< 0.001	<0.001	−	< −0.001	<0.001	< 0.001	<0.001	< 0.001
χ^2^*/gl*	15.637	3.299	2.572	2.049	−	4.141	2.649	1.239	2.809	1.807
*GFI*	0.981	0.974	0.948	0.959	−	0.965	0.966	0.981	0.980	0.965
*AGFI*	0.970	0.958	0.916	0.935	−	0.944	0.946	0.970	0.969	0.944
*NFI*	0.968	0.896	0.823	0.875	−	0.918	0.889	0.945	0.946	0.947
*NNFI*	0.960	0.898	0.840	0.906	−	0.914	0.902	0.985	0.952	0.967
*CFI*	0.970	0.924	0.881	0.930	−	0.936	0.927	0.989	0.964	0.976
*RMSEA* (CL 90%)	0.048 (0.045,0.052)	0.050 (0.041,0.059)	0.067 (0.051,0.083)	0.053 (0.037,0.069)	−	0.059 (0.050,0.068)	0.054 (0.042,0.066)	0.022 (0.000,0.040)	0.041 (0.032,0.050)	0.046 (0.029,0.063)
*SRMR*	0.034	0.043	0.059	0.046	−	0.041	0.045	0.033	0.032	0.045

Indices of fit: χ^2^, Minimum value of the discrepancy function optimized for maximum likelihood; df, degrees of freedom of the statistic χ^2^, p, probability of the statistic χ^2^ for H_0_: χ^2^, 0 having a tail (goodness of fit), χ^2^/df, relative chi-squared; GFI, goodness of fit index; AGFI, corrected goodness of fit index; NFI, normalized fit index; NNFI, non-normalized fit index; CFI, comparative fit index and RMSEA (90% CL); quadratic mean approximation error (estimation per interval with a confidence level of 90%) and SRMR, standardized mean quadratic error.

With regard to the CFA per country, the goodness of fit values for Spain were good for five of the eight indices (χ2/gl, GFI, AGFI, RMSEA, and SRMR), while the value for CFI was acceptable and the values for NFI and NNFI were close to those desired. In the case of Ukraine, the values were good for five of the eight indices (χ2/gl, GFI, AGFI, RMSEA, and SRMR), while the values for CFI, NFI, and NNFI were close to those desired. In the Moroccan sample, the values were good for four of the eight indices (χ2/gl, GFI, AGFI, RMSEA, and SRMR), while the NNFI and CFI values were acceptable and that for NFI was close to the desired value. With regard to the Bosnian sample, the values were good for four of the eight indices (GFI, AGFI, RMSEA, and SRMR) and acceptable for the χ2/gl, NFI, NNFI, and CFI. In the Mexican sample, the values were good for five of the eight indices (χ2/gl, GFI, AGFI, RMSEA, and SRMR), the NNFI and CFI values were acceptable, and the NFI was close to the desired value. All eight goodness of fit indices were good for the samples obtained from Colombia, Chile, and Brazil.

As mentioned, the internal consistency was checked through the McDonald coefficient (ω > 0.70; Raykov and Hancock, 2005), obtaining acceptable values for the Egalitarian Beliefs factor (ω ≥ 0.772) and the Sexist Beliefs factor (ω ≥ 0.70) but unacceptable values for the Perception of Sexist Relationships factor (ω < 0.70).

### Distribution of the factors of the SAGEFS

The distributions of EB, SB, and PSR, both in general and by gender, are shown in Table 3. The three factors have positive asymmetry or a long tail toward the right. None of them have a normal distribution (Table 4). Table 3, meanwhile, shows the descriptive statistics of the three factors of the SAGEFS separated into samples per country. In this particular case, the sample obtained for Iran was not considered for the PSR because data regarding that factor were not obtained.

**TABLE 3:** Descriptive statistics of the factors of the SAGEFS per country.

		*M*	*SD*	CL 95%		*Min*	*Max*
				*LI*	*LS*		
EB	Bosnia	20.88	4.415	20.59	21.17	6	30
	Ukraine	19.06	4.382	18.60	19.52	6	30
	Spain	24.89	4.702	24.58	25.19	6	30
	Morocco	24.36	4.531	23.90	24.82	9	30
	Mexico	23.01	4.685	22.63	23.40	6	30
	Colombia	24.09	4.467	23.69	24.49	6	30
	Chile	24.35	4.610	24.08	24.63	6	30
	Brazil	23.58	4.861	23.09	24.08	6	30
	Iran	16.76	4.416	16.49	17.03	6	30
SB	Bosnia	32.86	7.976	32.34	33.38	10	50
	Ukraine	30.28	6.859	29.56	31.00	11	48
	Spain	43.78	6.768	43.34	44.21	10	50
	Morocco	42.70	6.659	42.02	43.37	14	50
	Mexico	39.39	7.284	38.79	40.00	15	50
	Colombia	40.28	6.278	39.72	40.84	13	50
	Chile	41.77	6.492	41.38	42.16	10	50
	Brazil	41.80	6.304	41.16	42.44	14	50
	Iran	29.81	4.777	29.52	30.10	11	46
PSR	Bosnia	12.94	3.219	12.73	13.15	4	20
	Ukraine	12.70	2.643	12.43	12.98	6	20
	Spain	9.58	2.631	9.41	9.75	4	20
	Morocco	12.16	4.078	11.75	12.58	4	20
	Mexico	11.61	3.297	11.34	11.88	4	20
	Colombia	11.12	3.679	10.79	11.45	4	20
	Chile	11.81	3.559	11.60	12.02	4	20
	Brazil	13.05	3.250	12.72	13.38	4	20

M (CI 95%), arithmetic mean as regards accurate estimation and per interval with a confidence level of 95%; SD, standard deviation of sample; Min, minimum value; Max, maximum value; EB, egalitarian beliefs; SB, sexist beliefs; PSR, perception of sexist relationships.

**TABLE 4:** Descriptive statistics of the factors of the SAGEFS at a general level and by gender.

Statistic	EB	SB	PSR	MEB	FEB	MSB	FSB	MPSR	FPSR
*M*	22.14	37.79	11.75	21.49	22.77	36.27	39.28	11.16	12.20
	CL95%	CL95%	CL95%	CL95%	CL95%	CL95%	CL95%	CL95%	CL95%
	(22.00, 22.27)	(37.57, 38.00)	(11.66, 11.84)	(21.30, 21.67)	(22.58, 22.96)	(35.94, 36.57)	(38.97, 39.59)	(11.03, 11.29)	(12.07, 12.34)

M (CI 95%), arithmetic mean as regards its accurate estimation and per interval with a confidence level of 95%, SD, standard deviation of sample; Min, minimum value; Max, maximum value; KSL, Kolmogorov-Smirnov test with the correction for Lilliefors significance; EB, egalitarian beliefs; SB, sexist beliefs; PSR, perception of sexist relationships; MEB, male egalitarian beliefs; MSB, male sexist beliefs; MPSR, male perception of sexist relationships; FEB, female egalitarian beliefs; FSB, female sexist beliefs; FPSR, female perception of sexist relationships.

### Comparison of means by gender with respect to the three factors of the SAGEFS

Upon comparing the mean regarding the NSCS of the male and female students, a significant difference of t = −9.32, p < 0.001 was found. The mean for the female students was greater. The effect size of gender on the NSCI was small, g = 0.24. The mean of the female students was significantly greater than that of the male students regarding the NSCS, t = −14.04, p < 0.001, with a small effect size of gender on the NSCS, g = 0.36. The NR was significantly greater for the female students than for the male students, t = −10.76, p < 0.001. The effect size of gender on the NR was small, g = 0.30 (Table 5).

**TABLE 5:** Intergroup comparison depending on gender.

	Groups	Descriptive			Levene		Student			*g*
		*N*	*M*	*SD*	*F*	*p*	*t*	*df*	*p*	
EB	Male	3019	21.49	5.38	0.004	0.948	−9.32	6099	0.000	0.24
	Female	3082	22.77	5.37						
SB	Male	3019	36.27	8.44	0.196	0.658	−14.04	6099	0.000	0.36
	Female	3082	39.28	8.35						
PSR	Male	2479	11.16	3.25	29.58	0.000	−10.76	5040.82	0.000	0.30
	Female	2586	12.20	3.63						

N, number of cases; M, arithmetic mean; SD, standard deviation. Levene Test: F; contrast statistic, p; probability of a tail. Student’s t-test for independent samples: t, contrast statistic; df, degrees of freedom when df is < 6099 and p, probability of two tails; g, Hedges’ effect size statistic. EB, egalitarian beliefs; SB, sexist beliefs; PSR, perception of sexist relationships.

### Concurrent construct validity regarding perceived motor competence

The correlations of the absolute value with the three external validity criteria varied from 0.111 to 0.324. The MC accordingly correlated to a greater extent with the SB factor than with NSCI and PSR. The correlations among MC, EB, and PSR were, therefore, very similar but of the opposite sign (Table 6).

**TABLE 6:** Correlations between SAGEFS and external validity criterion with CL 95%.

External validity criterion	SAGEFS		
	EB	SB	PSR
MC	−0.111*	−0.324*	0.124*
	(−0.135, −0.086)	(−0.348, −0.300)	(0.095,0.153)

*p < 0.001. EB, egalitarian beliefs; SB, sexist beliefs; PSR, perception of sexist relationships; MC, perceived motor competence.

## Discussion

The present study focuses on the attitudes toward gender equality in soccer. The aim of this study was to adapt the Scale of Attitudes toward Gender Equality in Football in the School Context (**SAGEFS**) to the context of Spain, Ukraine, Morocco, Iran, Bosnia, Mexico, Colombia, Chile, and Brazil. The results shown above made it possible to obtain a valid and reliable instrument that permits the analysis of intercultural differences.

The lack of studies regarding gender equality in the context of schools was mentioned by Martínez-Galindo (2006) a decade and a half ago, and the interest in this subject has continued to grow at a social level. In the case of soccer, studies on gender equality are even more scarce, particularly if we consider that it is an aspect of Physical Education that is closely associated with male gender or boys (Valdivia-Moral et al., 2018). All of that stated above, therefore, provides significance to this study, since it shows the definition of a valid measurement instrument for something as important as soccer. Factors such as skills, beliefs, and previous experience regarding gender in Physical Education, and how they are promoted, have also been taken into account (Dragutinovic and Mitrovic, 2019; Reina et al., 2019). The scale presented herein sheds light on three dimensions, which can be described as follows.

### Egalitarian beliefs

This dimension is composed of a series of indicators that evidence the student’s favorable attitude toward girls playing soccer in an egalitarian manner. It signifies that the same importance is placed on matches played by girls and boys, that the same amount of time and resources should be invested, and that women’s soccer is considered to be a benefit to society in terms of reducing sexist beliefs. A negative view in this dimension would show an unfavorable attitude of the students towards the practice of football in an equal manner. This would imply that students consider that more resources should be invested in boys than in girls. On the other hand, they would consider that women’s football has little impact on society.

### Perception of sexist relationships

This indicates the degree to which the student perceives girls to be accepted in soccer, in the form of physical and psychological aggression.

The dimensions of this questionnaire were influenced by studies that promote attitudes toward equality Azorín (2017) and Garaigordobil and Durá (2005). With regard to the perception of sexist relationships, Azorín (2017) and Gil-Madrona et al. (2014), along with Gil-Madrona et al. (2017), have placed importance on the fact that boys should be more permissive in terms of allowing their female classmates to play this sport with them.

With regard to the evaluation of the psychometric properties of the instrument, the CFA provides evidence of its construct validity by obtaining a three-factor model with its residues of independent measures regarding both the sample as a whole and the samples obtained from each participating country. The fitness indicators were good and the original factorial structure was confirmed with appropriate factorial weights (λ’s > 0.40). It was, however, necessary to eliminate nine items in order to obtain better indicators.

In this respect, the CFA is similar to instruments like the CACEF created by Valdivia-Moral et al. (2015), which studies the coeducational beliefs of Physical Education teachers. The instrument in question was, however, validated within a population at Spanish schools and deals with different aspects of Physical Education. Other less recent instruments in the same line are those developed by Del-Castillo (2009) in Spain and Papaioannou (1998) in Greece.

The reliability of McDonald’s omega was acceptable for the Egalitarian Belief (ω ≥ 0.772) and Sexist Belief (ω ≥ 0.70) factors but not for the Perception of Sexist Relations factor. This may be related to the idiosyncratic characteristics of each country regarding the relationship between the gender when playing soccer. As stated by Borges and Branco (2019), this is owing to the fact that each country’s popular culture is molded into the different social relationships that appear in the context of schools. In this study, we, therefore, stress that the approach employed in this work brings together different cultures and religions in which the relationships between genders are different.

The analyses conducted led to the creation of a reduced version of the SAGEFS, which examines the attitudes toward gender in the context of schools by interpreting egalitarian and sexist beliefs, along with the perception of sexist relationships.

In conclusion, the results of the analysis of the different samples obtained for the SAGEFS evidence the presence of psychometric properties that are indispensable for the measurement of attitudes toward gender equality in the context of schools.

It is, however, necessary to carry out more psychometric studies in varied populations, in addition to analyzing the factorial invariance for country and gender. It would also be possible to carry out similar studies focused on the contents of Physical Education that are associated with gender, such as dance.

## Data availability statement

The datasets presented in this article are not readily available because all requests to access the datasets should be directed to pedro.gil@uclm.es.

## Ethics statement

The studies involving humans were approved by the Comisión de convivencia y ética escolar del CRA “Calar del Mundo”. Castilla-La Mancha. Universidad de Castilla-La Mancha. The studies were conducted in accordance with the local legislation and institutional requirements. Written informed consent for participation in this study was provided by the participants’ legal guardians/next of kin.

## Author contributions

LM-H: Methodology, Investigation, Writing—original draft, Formal analysis, Validation. PG-M: Writing—original draft, Writing—review and editing, Methodology, Investigation, Project administration, Supervision. DZ-G: Conceptualization, Writing—original draft, Validation, Formal analysis, Methodology, Writing—review and editing. JS-P: Writing—original draft, Formal analysis, Methodology, Validation, Conceptualization, Data curation, Writing—review and editing. JP-C: Writing—review and editing, Data curation, Formal analysis, Investigation, Validation. EF-F: Writing—review and editing, Data curation, Investigation, Project administration, Supervision. MG-V: Writing—review and editing, Data curation, Investigation, Conceptualization, Formal analysis. RP: Data curation, Investigation, Writing—review and editing, Project administration, Supervision. FM-C: Conceptualization, Data curation, Investigation, Project administration, Writing—review and editing. NG-S: Supervision, Conceptualization, Data curation, Investigation, Writing—review and editing. SM-C: Project administration, Data curation, Investigation, Supervision, Writing—review and editing. MC-R: Formal analysis, Investigation, Supervision, Writing—review and editing.
